# Quercetin treatment protects the Achilles tendons of rats from oxidative stress induced by hyperglycemia

**DOI:** 10.1186/s12891-022-05513-4

**Published:** 2022-06-10

**Authors:** Tomoya Yoshikawa, Yutaka Mifune, Atsuyuki Inui, Hanako Nishimoto, Kohei Yamaura, Shintaro Mukohara, Issei Shinohara, Ryosuke Kuroda

**Affiliations:** Department of Orthopaedic Surgery, Kobe University Graduate School of Medicine, 7-5-1 Kusunoki-cho, Chuo-ku, Kobe, Hyogo 650-0017 Japan

**Keywords:** Quercetin, Oxidative stress, Antioxidant effect, Diabetic tendinopathy, Rat diabetic model

## Abstract

**Background:**

Quercetin, a flavonoid abundantly in vegetables and fruits, exerts antioxidant and anti-inflammatory effects. We investigated the protective effects of quercetin against oxidative stress in the Achilles tendons of diabetic rats.

**Methods:**

Cells were collected from the Achilles tendons of Sprague–Dawley rats and cultured under four conditions: regular glucose (RG) without quercetin (Quer-), RG with quercetin (Quer +), high-glucose (HG) Quer-, and HG Quer + . The expression of genes related to NADPH oxidase (NOX) and inflammation, reactive oxygen species accumulation, and apoptosis rates was analyzed. Additionally, diabetic rats were divided into two groups and subjected to quercetin (group Q) or no quercetin (group C) treatment. Histological evaluation and expression analysis of relevant genes in the Achilles tendon were performed.

**Results:**

In rat tendon-derived cells, the expression of *Nox1*, *Nox4*, and *Il6*; reactive oxygen species accumulation; and apoptosis rates were significantly decreased by quercetin treatment in the HG group. The collagen fiber arrangement was significantly disorganized in the diabetic rat Achilles tendons in group C compared with that in group Q. The mRNA and protein expression levels of NOX1 and NOX4 were significantly decreased upon quercetin treatment. Furthermore, the expression of *Il6*, type III collagen, *Mmp2*, and *Timp2* was significantly decreased, whereas that of type I collagen was significantly increased in group Q compared with that in group C.

**Conclusions:**

Quercetin treatment decreases NOX expression and thus exerts antioxidant and anti-inflammatory effects in the Achilles tendons of diabetic rats. Quercetin treatment may be effective against diabetic tendinopathy.

## Background

Patients with diabetes mellitus (DM) are at a greater risk of developing musculoskeletal disorders compared to patients without DM; additionally, patients with DM show impaired tendon healing ability [1, 2]. Lee et al. reported that DM is associated with an increase in the prevalence of general shoulder pathologies, such as shoulder pain and rotator cuff tendinitis; thickening of the rotator cuff tendons has also been observed [3]. Diabetes is also associated with a higher risk of tendinitis [4], with potential functional changes [5, 6] and structural abnormalities, including the loss of collagen organization [7] or thickening [4, 5, 8] and calcification [9], all of which may increase the risk of tendon rupture [10]. The severity of DM is associated with worsening effects in the context of tendinitis. For example, degeneration of the Achilles tendon was more severe in tendinopathic patients with DM than in patients without DM [11]. Additionally, musculoskeletal disorders of the hands and shoulders are approximately four-fold more frequent in patients with diabetes than in patients without diabetes [12].

DM is characterized by hyperglycemia [13]. Excessive generation of oxidative stress induced by hyperglycemia has been reported to cause tissue damage and organ dysfunction [14]. Under high-glucose conditions, the levels of intracellular reactive oxygen species (ROS), as the main agents of oxidative stress, are increased [15]. Increased levels of ROS can induce damage to DNA, RNA, and proteins as well as changes in the levels of antioxidant enzymes, ultimately leading to cell and tissue damage [16, 17]. Previous studies showed that the primary source of ROS is nicotinamide adenine dinucleotide phosphate oxidase (NOX); activation of this enzyme leads to increased ROS production [18–20]. For instance, cultured aortic smooth muscle cells and endothelial cells under hyperglycemic conditions showed increased ROS production via protein kinase C-dependent activation of NOX [21].

The effectiveness of intensive therapy in diabetic complications, such as tendinopathy, remains limited; thus, new approaches are needed to prevent the progression of diabetic complications. Most anti-diabetes medications have several negative side effects, including hypoglycemia, weight gain, ketoacidosis, skeletal fragility, and heart failure [22–24]. In contrast, antioxidant therapy has been reported to prevent oxidative damage in patients with diabetes [25].

Natural phenolic compounds have been reported to affect hypertension by inhibiting the expression of NOX and consequent decrease in ROS production [26]. Quercetin, a polyphenol in the group of flavonoid compounds found in vegetables, fruits, leaves, and seeds [27], is a multifunctional agent with antioxidant [25, 28, 29], anti-inflammatory [30], anticancer [31], and anti-insulin resistance [32] effects. Quercetin has been shown to exert protective effects on different types of cells, including cardiomyocytes, neurons, and testes, kidney, and liver cells following ischemia/reperfusion injury [33–35]. However, its antioxidant functions in tendon-derived cells and tendon tissues remain unclear. Therefore, in this study, we evaluated the tendon-protective effects of quercetin against oxidative stress induced by hyperglycemia.

## Methods

All animal experiments were conducted under the approval and guidance of the Animal Care and Use Committee of our institution. The experiment was performed according to the ARRIVE guidelines. Male Sprague–Dawley rats (8 weeks old) were used in this study. The rats were housed in standard cages and had unrestricted access to food, water, and activities under a regular light–dark cycle.

### *In vitro* experiments

#### Cell culture

Ten 8-week-old normal Sprague–Dawley rats were used for the *in vitro* study. Their Achilles tendons were excised and washed twice with phosphate-buffered saline. The tendon tissues were cut into small pieces of approximately 1.5–2.0 mm^3^, and several pieces were cultured in Dulbecco’s modified Eagle’s medium (DMEM; HyClone, Logan, UT, USA) supplemented with 10% fetal bovine serum (Cansera, Toronto, Canada), 100 U/mL penicillin, and 100 µg/mL streptomycin. The explants were incubated at 37 °C in a humidified atmosphere of 5% CO_2_/95% air. The cells from the tendons were subcultured after trypsin digestion; the medium was changed every 3–5 days. Cells at passages 2–3 were used for experiments.

#### Cell proliferation assay

Cell proliferation was measured in a water-soluble tetrazolium salt (WST) assay using the Cell Counting Kit-8 (Dojindo, Kumamoto, Japan) to confirm the toxicity of quercetin and determine the optimal concentration of quercetin. All wells in 96-well plates were seeded with 5,000 cells in 100 μL DMEM. The cells were exposed to DMEM with a constant glucose concentration (6 mM) and six different quercetin concentrations (0, 1, 10, 20, 50, and 100 μM: the replicate number was 3, *n* = 10 per group); the cells were incubated at 37 °C in a 5% CO_2_ atmosphere for 48 h. For the WST assay, 10 μL of WST was added to each well, and the plates were incubated for an additional 4 h at 37 °C in a 5% CO_2_ atmosphere. The conversion of WST to formazan was spectrophotometrically measured at 450 nm.

#### Experimental groups

Achilles tendon-derived cells were seeded onto 12-well culture plates at a density of 1 × 10^5^ cells per well and incubated in DMEM with two different glucose concentrations, 6 mM in the regular-glucose (RG) group and 33 mM in the high-glucose (HG) group without fetal bovine serum to avoid overgrowth. Quercetin (Cayman Chemical Company, Ann Arbor, MI, USA) was dissolved in dimethyl sulfoxide to obtain a 100 mM stock solution and diluted to produce a quercetin concentration of 20 µM; quercetin was added to cells immediately after seeding. Four different groups were analyzed: a) RG group without quercetin (RG Quer-), b) RG group with quercetin (RG Quer +), c) HG group without quercetin (HG Quer-), and d) HG group with quercetin (HG Quer +).

#### Quantitative reverse transcription polymerase chain reaction analysis

After 48 h of incubation, total RNA was extracted from each group of cells using an RNeasy Mini Kit (Qiagen, Hilden, Germany). Total RNA was reverse-transcribed into single-strand cDNA using a high-capacity cDNA reverse transcription kit (Applied Biosystems, Foster City, CA, USA). Real-time polymerase chain reaction (PCR) was performed in triplicate on an Applied Biosystems 7900HT fast real-time PCR system and SYBR Green reagent (Applied Biosystems) to analyze the mRNA expression levels of *Nox1*, *Nox4*, and interleukin 6 (*Il6*). The primer sequence was the same as in previous reports [15] (Table 1) and obtained from Thermo Fisher Scientific Inc (Waltham, MA, USA). Gene expression was normalized to that the mRNA level of *Gapdh*, and the stability of *Gapdh* expression was tested under all conditions. The results are expressed log_2_-fold changes relative to those of control cells, as per the 2^−ΔΔCt^ method (the replicate number was 3, *n* = 10 per group).

**Table 1:** Primer sequences used for qRT-PCR analysis

*Gene*	Oligonucleotide sequence
*Nox1*	Forward 5′ GTGGCTTTGGTTCTCATGGT 3' Reverse 5' TGAGGACTCCTGCAACTCCT 3′
*Nox4*	Forward 5′ GGGCCTAGGATTGTGTTTGA 3' Reverse 5' CTGAGAAGTTCAGGGCGTTC 3′
*Il6*	Forward 5′ GGTCTTCTGGAGTTCCGTTTC 3' Reverse 5' GGTCTTGGTCCTTAGCCACTC 3′
Type I collagen	Forward 5′ TGGAGACAGGTCAGACCTG 3′ Reverse 5′ TATTCGATGACTGTCTTGCC 3′
Type III collagen	Forward 5′ TAAAGGGTGAACGGGGCAGT 3′ Reverse 5′ ACGTTCCCCATTATGGCCAC 3′
*Mmp2*	Forward 5′ GGAAGCATCAAATCGGACTG 3′ Reverse 5′ GGGCGGGAGAAAGTAGCA 3′
*Timp2*	Forward 5′ GGACACGCTTAGCATCACCCAGA 3′ Reverse 5′ GTCCATCCAGAGGCACTCATCC 3′
*Gapdh*	Forward 5′ GGTGGTCTCCTCTGACTTCAACA 3′ Reverse 5′ GTTGCTGTAGCCAAATTCGTTGT 3′

*NOX* Nicotinamide adenine dinucleotide phosphate oxidase, *IL* Interleukin, *MMP* Matrix Metalloproteinase, *TIMP* Tissue inhibitor of matrix metalloproteinase, *GAPDH* Glyceraldehyde 3-phosphate dehydrogenase

#### ROS measurements

In total, 1 × 10^5^ cells per well were seeded into 1 mL of DMEM in 12-well plates and incubated at 5% CO_2_ and 37 °C for 48 h. The accumulation of intracellular ROS in Achilles tendon-derived cells was detected using the oxidation-sensitive fluorescent probe 2′7′-dichlorofluorescin diacetate (DCFH–DA) with a Total ROS/Superoxide Detection Kit (Enzo Life Sciences, Farmingdale, NY, USA), according to the manufacturer’s protocol. Briefly, Achilles tendon-derived cells were incubated with a final DCFH–DA concentration of 10 µM for 60 min at 37 °C in the dark, washed three times with phosphate-buffered saline, trypsinized, and resuspended. ROS-positive cells and 2-(4-amidinophenyl)-1H-indole-6-carboxamidine (DAPI)-positive cells in four rectangular areas (0.75 × 1.0 mm) were counted on each well, and their average values were calculated. The cells were manually counted by two blinded investigators. The rate of ROS-positive cells (number of ROS-positive nuclei/number of DAPI-positive nuclei) was determined as the mean of the values from the four areas (the replicate number was 3, *n* = 10 per group).

#### Immunofluorescence staining to analyze apoptotic cells

Nuclear fragmentation in fixed cells (4% paraformaldehyde/phosphate-buffered saline) was detected via terminal deoxynucleotidyl transferase dUTP nick end labeling (TUNEL) staining using an APO-DIRECT™ Kit (Phoenix Flow Systems, San Diego, CA, USA) according to the manufacturer’s protocol; DAPI was used to counterstain the nuclei. Apoptosis-positive cells and DAPI-positive cells in four rectangular areas (0.75 × 1.0 mm) were counted on each well, and their average values were calculated. The cells were manually counted by two blinded investigators. The rate of apoptosis-positive cells (number of apoptosis-positive nuclei/number of DAPI-positive nuclei) was determined as the mean value of the four areas (the replicate number was 3, *n* = 10 per group).

### *In vivo* experiments

#### Type 1 diabetes rat model and experimental groups

Type 1 DM was induced in Sprague–Dawley rats via treatment with streptozotocin (STZ; 65 mg/kg; Sigma-Aldrich) [15, 36, 37]; 20 male 8-week-old rats were used in this *in vivo* study. All rats injected with STZ became diabetic by 7 days after STZ injection. Their mean blood glucose level was 451.9 ± 61.8 mg/mL (mean ± standard deviation), whereas that of healthy control rats was < 150 mg/dL [32]. The rats were randomly divided into two groups and subjected to quercetin (group Q) or no quercetin (group C) treatment (*n* = 10 in each group). At two weeks after STZ administration, quercetin was administered intraperitoneally at a dose of 50 mg/kg every other day [38] for 4 weeks. In group C, the solvent 10% dimethyl sulfoxide was administered in the same manner. Thereafter, the Achilles tendons were harvested after all rats were anesthetized with isoflurane and stored at -80 °C for further analysis; the right Achilles tendon was used for histological and immunohistochemistry evaluations, and the left tendon was used for quantitative reverse transcription (qRT)-PCR analysis.

#### Achilles tendon histology and immunohistochemistry

Each of the 20 right Achilles tendons collected from diabetic rats administered quercetin or no quercetin was subjected to histological and immunohistochemistry analyses. Frozen long-axis sections of Achilles tendons embedded in optimal cutting temperature compound (Sakura Finetek USA, Inc., Torrance, CA, USA) were sequentially sectioned into 7-μm-thick sections and fixed using 10% phosphate-buffered paraformaldehyde for 15 min at room temperature of 20–25 °C. The fiber structure and arrangement, nuclear morphology, and regional variations of tendon cells were evaluated using hematoxylin and eosin (H&E) staining [39]. Each variable was scored from 0 to 3, where 0 = normal, 1 = slightly abnormal, 2 = abnormal, and 3 = significantly abnormal. H&E-stained Achilles tendons were graded in five randomly selected optical fields per histological section. Two blinded investigators analyzed each field. Additionally, to evaluate the expression of NOX, anti-NOX1 (ab131088; rabbit polyclonal to NOX1, Abcam, Cambridge, UK) and anti-NOX4 antibodies (ab133303; rabbit monoclonal to NOX4, Abcam) were used for immunohistochemistry staining. Briefly, the sections were incubated with proteinase for 10 min, treated with 3% hydrogen peroxide (Wako Pure Chemical Industries, Osaka, Japan) to inhibit endogenous peroxidase activity, and incubated with anti-NOX1 or anti-NOX4 antibodies (both 1:100) overnight at 4 °C. The sections were incubated with the respective peroxidase-labeled secondary antibodies (anti-rabbit IgG polyclonal antibody, Nichirei Bioscience, Tokyo, Japan) for 30 min at room temperature. The signals for NOX1 and NOX4 were detected via the formation of a brown color following incubation with the peroxidase substrate 3,3′-diaminobenzidine (Nichirei Bioscience). The sections were counterstained with hematoxylin and analyzed under a microscope (BZ-X710, Keyence, Osaka, Japan). The percentage of NOX-positive tendon cells per field counted manually by two blinded investigators was averaged from five randomly selected fields per tissue section [15].

#### qRT-PCR analysis

The left Achilles tendons of diabetic rats treated with quercetin or not were used for qRT-PCR analysis. Briefly, the tendons were cut into small pieces, carefully isolated from the connective tissue contaminants, and minced; the tissues were then enzymatically digested with type II collagenase for subsequent RNA isolation [40]. Total RNA was extracted using an RNeasy Mini Kit. Reverse transcription into single-strand cDNAs and qRT-PCR were performed as described previously. The mRNA expression levels of *Nox1*, *Nox4*, *Il6*, type I collagen, type III collagen, matrix metalloproteinase 2 (*Mmp2*), and tissue inhibitor of matrix metalloproteinase 2 (*Timp2*) were determined using the primer pairs listed in Table 1. The results are expressed log_2_-fold changes relative to those of control group (group C).

### Statistical analysis

All data are expressed as the mean ± standard deviation (SD). All statistical analyses were performed using SPSS, version 27.0 (SPSS, Inc., Chicago, IL, USA). Significant differences between groups were detected using one-way analysis of variance (ANOVA) or independent *t*-test. Post-hoc analysis was performed using the Fisher’s protected least significant difference test. Statistical significance was set at *p* < 0.05.

## Results

### *In vitro* experiments

#### Cell proliferation assay

**Fig. 1:**
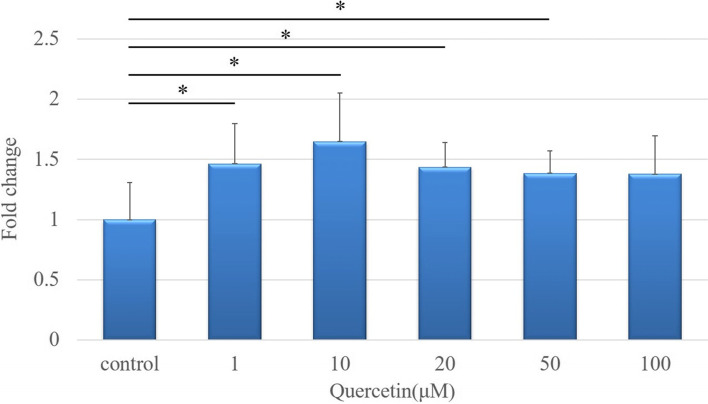
*In vitro* proliferation of tenocytes assessed using a water-soluble tetrazolium salt (WST)-based assay. Cells were cultured in DMEM with a constant glucose concentration (6 mM) in the presence of six different quercetin concentrations (0, 1, 10, 20, 50, and 100 μM) for 48 h. Data are expressed as the mean ± SD. One-way ANOVA was used to determine significant differences: *p < 0.05. *n* = 10 per group

The WST assay showed that the proliferation of cells cultured in the presence of 1, 10, 20, and 50 µM quercetin for 48 h was significantly higher than that of control cells (*p* < 0.05). The relative cell proliferation fold-changes are shown in Fig. 1. Importantly, none of the quercetin doses used showed cytotoxic effects on tenocytes (Fig. 1). However, because quercetin concentrations > 50 µM have been reported to have toxic effects [25], we used a quercetin dose of 20 µM.

#### qRT-PCR analysis

**Fig. 2:**
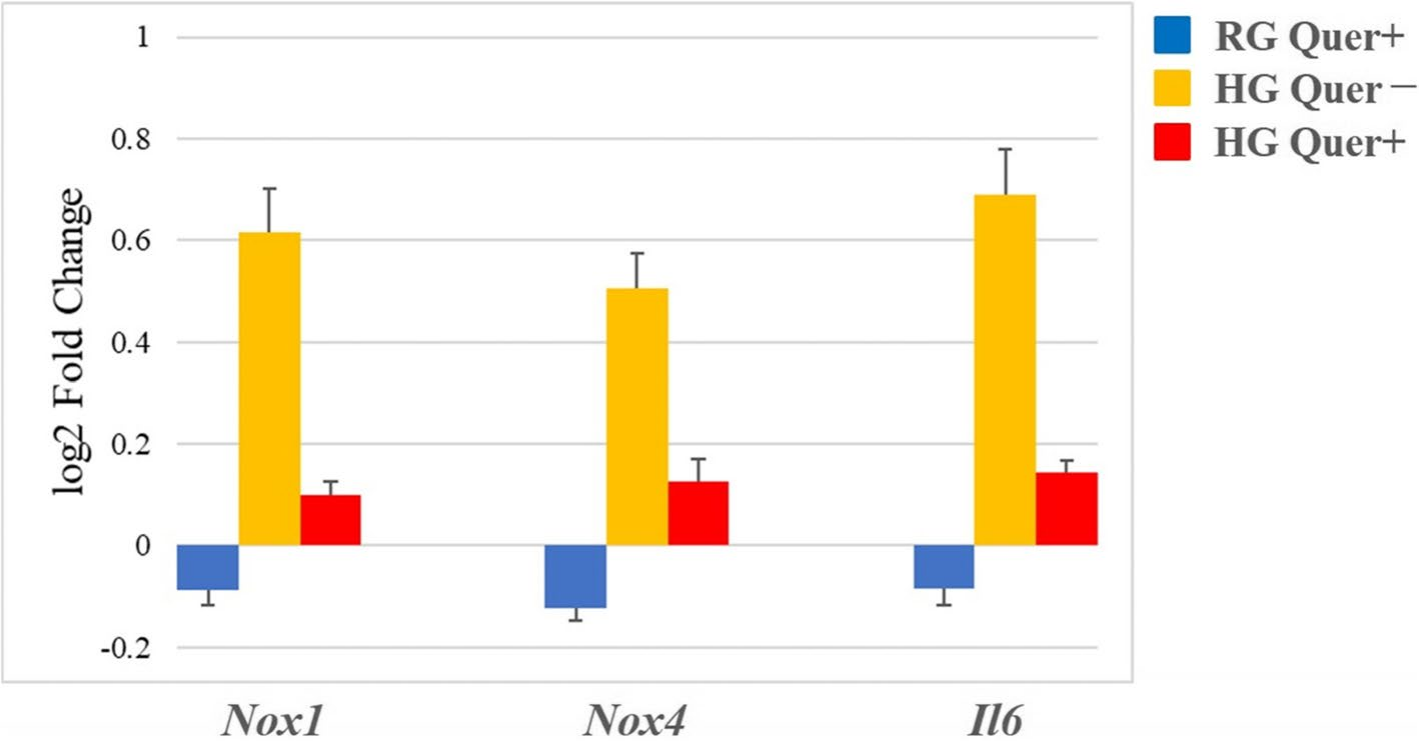
Quercetin treatment prevents upregulation of the expression of *Nox1*, *Nox4*, and *Il6* under HG conditions. The mRNA expression levels of these three genes were analyzed using qRT-PCR. Data are expressed as log_2_-fold changes normalized by the expressed transcripts of control cells (RG, Quer-). One-way ANOVA was used to determine significant differences: *p < 0.05. *n* = 10 per group. NOX, nicotinamide adenine dinucleotide phosphate oxidase; IL, interleukin; RG, regular-glucose; HG, high-glucose; Quer, quercetin

The mRNA expression levels of *Nox1*, *Nox4*, and *Il6* in the HG Quer- group were significantly higher than those in both the RG Quer- (*p* < 0.05) and RG Quer + groups (*p* < 0.05) (Fig. 2). Importantly, treatment with quercetin under HG conditions restored the mRNA expression levels of *Nox1*, *Nox4*, and *Il6* (expression was significantly lower in the HG Quer + group than that in the HG Quer- group (*p* < 0.05); Fig. 2). There was no significant difference between the expression levels of genes in the RG Quer- and RG Quer + groups (Fig. 2).

#### ROS measurements

**Fig. 3:**
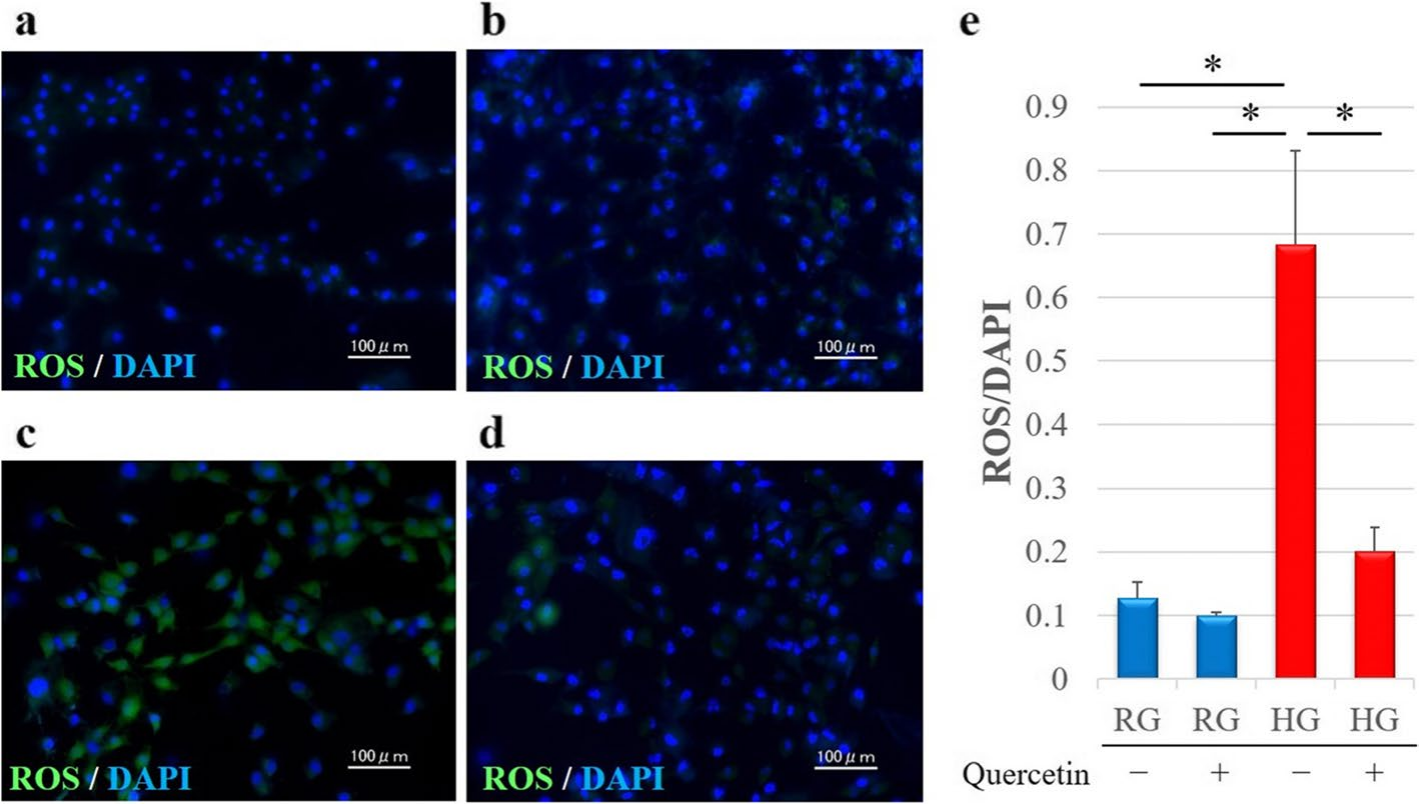
Representative immunofluorescence images of the detection of ROS levels in cells incubated in the presence of regular or high glucose with or without quercetin. **a** RQ Quer- group; **b** RG Quer + group; **c** HG Quer- group; **d** HG Quer + group. DCFH–DA staining (green) indicates accumulation of ROS in tenocytes; the nuclei are counterstained with DAPI (blue). **e** Relative quantification of ROS-positive cells. ROS-positive cells and DAPI-positive cells in four rectangular areas (0.75 × 1.0 mm) were counted on each slide and their average values were calculated. The rate of ROS-positive cells (number of ROS-positive nuclei/number of DAPI-positive nuclei) is shown as the mean of the four areas. Data are expressed as the mean ± SD. One-way ANOVA was used to determine significant differences: *p < 0.05. *n* = 10 per group. RG, regular-glucose; HG, high-glucose; ROS, reactive oxygen species; DAPI, 4′,6-diamidino-2-phenylindole

To determine the real *in vitro* oxidative context, intracellular ROS levels were detected via DCFH–DA staining; the cytoplasm of ROS-positive cells was stained as green (Fig. 3a–d). The results of quantitative analysis of ROS-positive cells are shown in Fig. 3e. The rate of ROS-positive cells in the HG Quer- group was significantly higher than that in the RG Quer- group (*p* < 0.05) and RG Quer + group (*p* < 0.05) (Fig. 3e). Additionally, in agreement with the qRT-PCR results, treatment with quercetin significantly affected ROS accumulation under HG conditions; ROS levels in the HG Quer + group were significantly lower than those in the HG Quer- group (*p* < 0.05). In contrast, ROS levels did not significantly differ in the RG Quer- and RG Quer + groups (Fig. 3e).

#### Immunofluorescence staining to analyze apoptotic cells

**Fig. 4:**
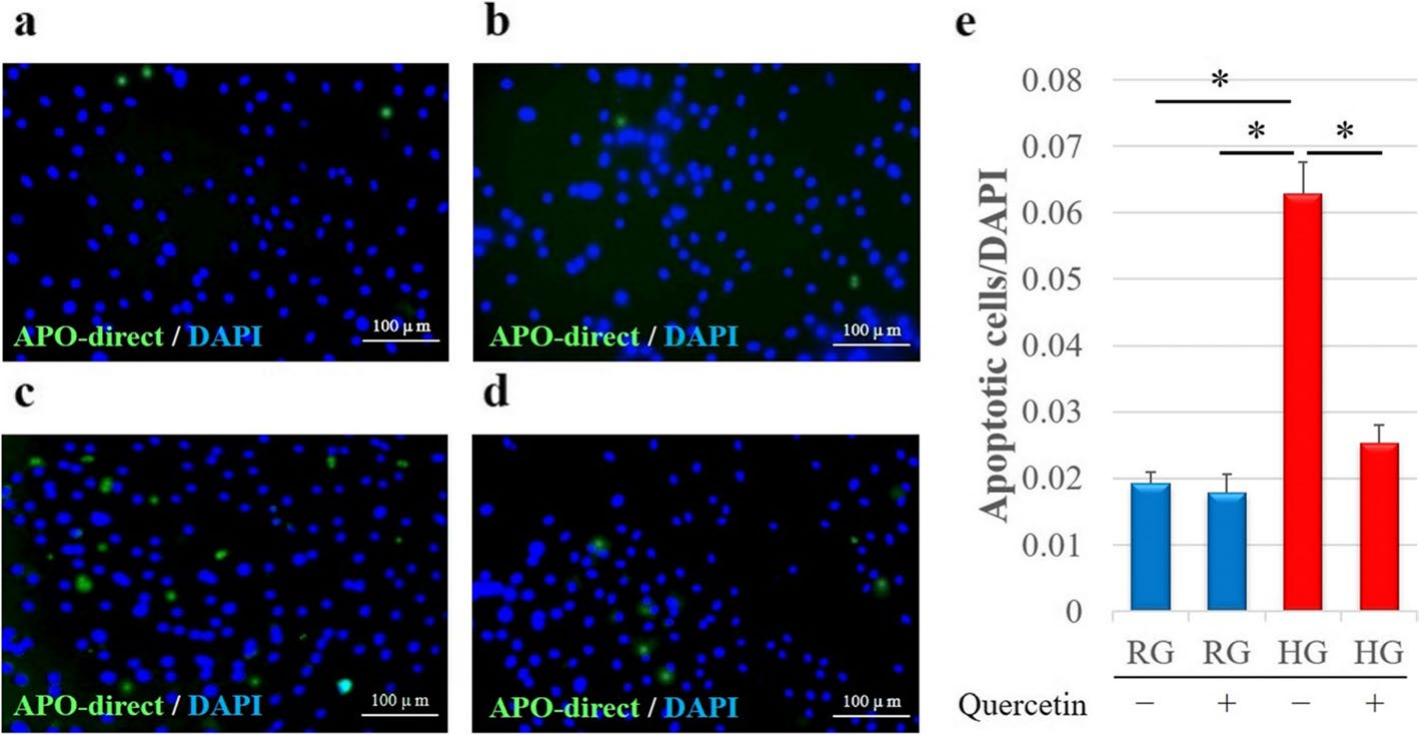
Representative immunofluorescence images of the detection of nuclear fragmentation (suggestive of apoptosis) in cells incubated in the presence of regular or high glucose with or without quercetin. **a** RQ Quer- group; **b** RG Quer + group; **c** HG Quer- group; **d** HG Quer + group. The TUNEL staining (green) highlights the apoptotic cells in each group. **e** Relative quantification of apoptotic cells. The number of apoptotic cells was analyzed as the fluorescence intensity normalized to cell number. Apoptosis-positive cells and DAPI-positive cells in four rectangular areas (0.75 × 1.0 mm) were counted in each slide, and their average values were calculated. The rate of apoptosis-positive cells (number of apoptosis-positive nuclei/number of DAPI-positive nuclei) is shown as the mean of the four areas. Data are expressed as the mean ± SD. One-way ANOVA was used to determine significant differences: *p < 0.05. *n* = 10 per group. RG, regular-glucose; HG, high-glucose; DAPI, 4′,6-diamidino-2-phenylindole

Next, apoptotic cells were detected via TUNEL staining; nuclear fragmentation of apoptotic cells is stained as green (Fig. 4a–d). The results of quantitative analysis of apoptotic cells are depicted in Fig. 4e. Interestingly, the ratio of apoptotic cells in the HG Quer- group was significantly higher than that in the RG Quer- (*p* < 0.05) and RG Quer + groups (*p* < 0.05) (Fig. 4e). Quercetin prevented the effect of high glucose levels on the apoptosis of tenocytes; the ratio of apoptotic cells in the HG Quer + group was significantly lower than that in the HG Quer- group (*p* < 0.05) (Fig. 4e). No significant differences were observed between the two RG groups (Fig. 4e).

### *In vivo* experiments

#### Achilles tendon histology and immunohistochemistry

**Fig. 5:**
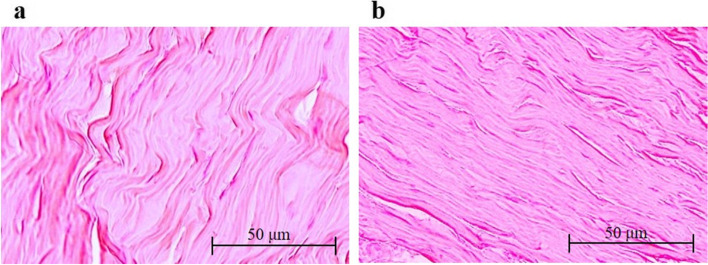
Achilles tendon histology. Hematoxylin and eosin staining of the Achilles tendons of diabetic rats treated with or without quercetin for four weeks. The collagen fiber arrangement was significantly disorganized in the tendons of diabetic rats in the control group (**a**, group C) compared with that in the tendons of diabetic rats treated with quercetin (**b**, group Q)

Histological evaluation of the Achilles tendons of diabetic rats in groups Q and C treatment revealed that the fiber arrangement was significantly more abnormal in the latter (0.88 ± 0.56 *versus* 0.46 ± 0.50, respectively, *p* < 0.05) (Table 2 and Fig. 5). There were no significant differences in the fiber structure (group C 0.78 ± 0.62, group Q 0.64 ± 0.56), nuclei roundness (group C 0.64 ± 0.53, group Q 0.54 ± 0.50), or regional variations in cellularity (group C 0.40 ± 0.50, group Q 0.32 ± 0.47) between the two groups (Table 2). Tenocytes from both groups showed flattened or spindle-shaped nuclei arranged in rows between the collagen fibers, and few rounded nuclei were observed (Fig. 5).

**Table 2:** Hematoxylin and eosin staining: tendon pathological scores

	Group C Mean (SD)	Group Q Mean (SD)	*P*-value
Fiber structure	0.78 (0.62)	0.64 (0.56)	0.12
Fiber arrangement	0.88 (0.56)	0.46 (0.50)	< 0.001*
Rounding of the nuclei	0.64 (0.53)	0.54 (0.50)	0.17
Regional variations in cellularity	0.40 (0.50)	0.32 (0.47)	0.2

Each variable was scored on a scale of 0–3, where 0 = normal, 1 = slightly abnormal, 2 = abnormal, and 3 = significantly abnormal. Achilles tendons stained with hematoxylin and eosin were graded in five optical fields randomly selected for each histological section. Data are expressed as the mean ± SD. The independent *t-*test was used to determine significant differences: *p < 0.05. *n* = 10 rats in the control group (group C), and *n* = 10 rats in the quercetin treatment group (group Q)

**Fig. 6:**
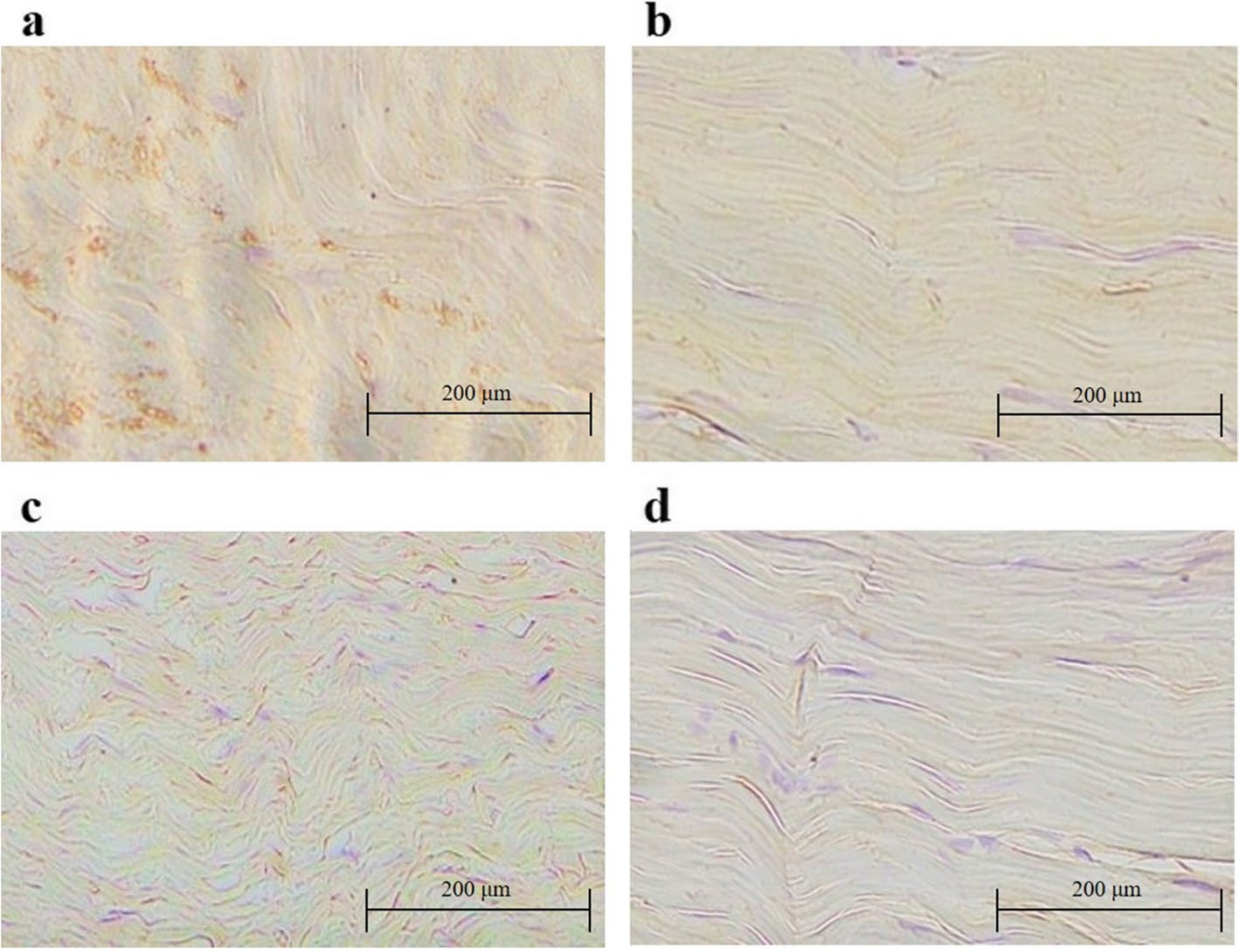
Immunohistochemistry staining to detect the expression of NOX 1 (**a**, **b**) and NOX4 (**c**, **d**) in the Achilles tendons of diabetic rats. The brown-stained cells represent NOX-positive cells. Decreased expression of NOX1 and NOX4 was observed in the Achilles tendons of diabetic rats treated with quercetin (**b**, **d**, group Q) compared that in the Achilles tendons of control diabetic rats (**a**, **c**, group C). NOX, nicotinamide adenine dinucleotide phosphate oxidase

**Fig. 7:**
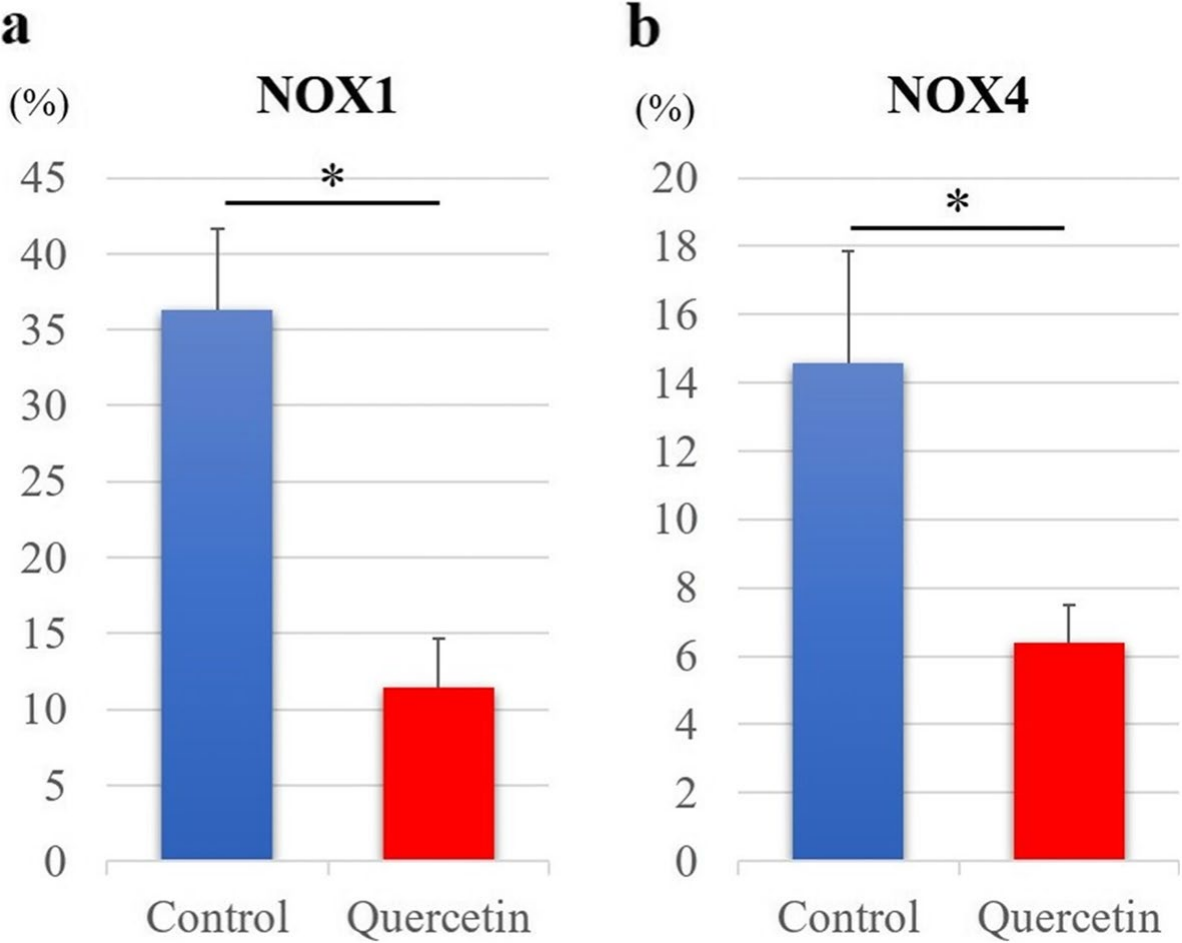
Quantitative analysis of cells expressing NOX1 (**a**) and NOX4 (**b**). The percentage of NOX-positive tendon cells per field was averaged from five randomly selected fields per tissue section. Data are expressed as the mean ± SD. The independent *t*-test was used to determine significant differences: *p < 0.05. *n* = 10 per group. NOX, nicotinamide adenine dinucleotide phosphate oxidase

Importantly, immunohistochemistry staining of NOX revealed that the expression levels of NOX1 and NOX4 were significantly decreased in tenocytes of rats treated with quercetin (group Q *versus* group C; Fig. 6). In line with this finding, the percentages of both NOX1-positive cells (group C 36.3 ± 5.31, group Q 11.5 ± 3.17, *p* < 0.001) and NOX4-positive cells (group C 14.6 ± 3.25, group Q 6.41 ± 1.10, *p* < 0.001) were significantly lower in the Achilles tendons of rats from group Q than in those of rats from group C (Fig. 7).

#### qRT-PCR analysis

**Fig. 8:**
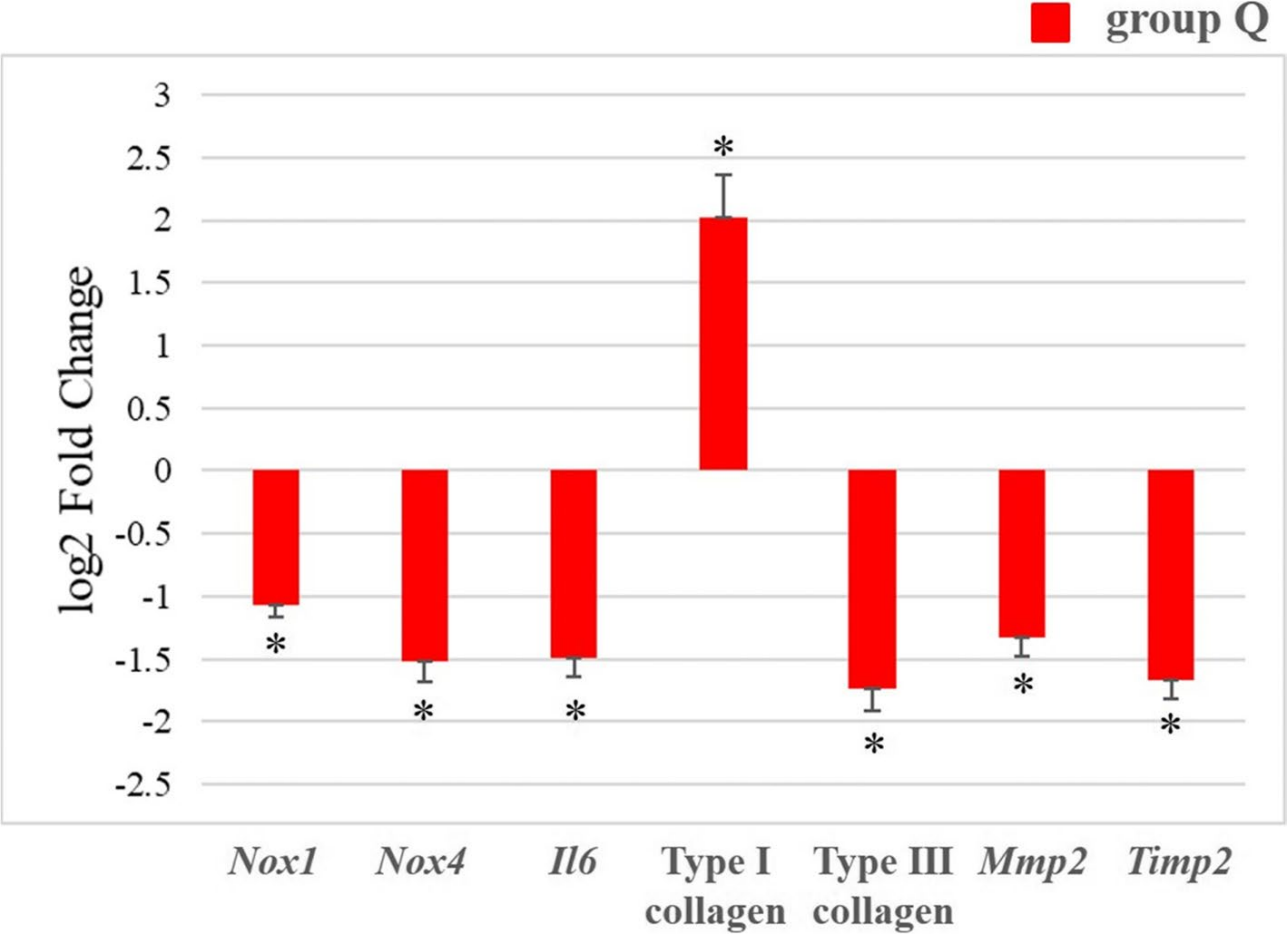
mRNA expression levels of *Nox1*, *Nox4*, *Il6*, type I collagen, type III collagen, *Mmp2*, and *Timp2* in the Achilles tendons of diabetic rats subjected to quercetin or no quercetin treatment analyzed using qRT-PCR. Data are expressed as log2-fold changes normalized by the expressed transcripts of control group (group C). The independent *t*-test was used to determine significant differences: *p < 0.05. *n* = 10 per group. NOX, nicotinamide adenine dinucleotide phosphate oxidase; IL, interleukin; MMP, matrix metalloproteinase; TIMP, tissue inhibitors of matrix metalloproteinase

The mRNA expression levels of *Nox*, *Il6*, type I collagen, type III collagen, *Mmp2*, and *Timp2* in the Achilles tendons of rats in the two groups were analyzed. In agreement with the *in vitro* findings, the mRNA expression levels of *Nox1*, *Nox4*, and *Il6* were significantly lower in group Q animals than in group C animals (*p* < 0.05; Fig. 8). Additionally, the mRNA expression levels of type III collagen, *Mmp2*, and *Timp2* were significantly lower and those of type I collagen were significantly higher in group Q than in group C (*p* < 0.05) (Fig. 8).

## Discussion

The aim of this study was to investigate the tendon-protective effect of quercetin against oxidative stress caused by hyperglycemia. This study revealed that administration of quercetin suppresses *Nox1*, *Nox4*, and *Il6* mRNA expression under HG conditions *in vitro*; these findings were confirmed *in vivo* in the Achilles tendons of diabetic rats treated with quercetin. Moreover, *in vivo*, immunostaining of NOX1 and NOX4 decreased in tenocytes from the Achilles tendons of diabetic rats treated with quercetin. Quantitative analysis of ROS production revealed that quercetin treatment reduced ROS production under hyperglycemic conditions. These results highlight the antioxidant and anti-inflammatory roles of quercetin in rat tendons exposed to HG conditions.

Quercetin, one of the most well-known flavonoids in the human diet [41], has attracted increasing attention because of its antioxidant [25, 28, 29], anti-inflammatory [30], anticancer [31, 42] anti-obesity [31, 43], anti-insulin resistance [32] antiviral [44], and antibacterial [45] effects. In fact, the antioxidant effects of quercetin have been demonstrated to play an important role in the prevention and treatment of various diseases, such as osteoporosis, cancer, and lung and cardiovascular diseases [33]. Quercetin exerts antioxidant effects primarily by affecting glutathione, enzyme activity, signaling pathways, and ROS triggered by environmental and toxic factors [28]. Additionally, quercetin has been reported to downregulate NOX expression and prevent endothelial dysfunction in spontaneously hypertensive rats [46]. Yousefian et al. reported that natural compounds, including quercetin, inhibit NOX expression and ROS production and improve hypertension through the formation of a stable radical from ROS-derived NOX and prevention of the assembly of NOX subunits [26].

Studies have shown that hyperglycemia induces oxidative stress and cytokine production, resulting in inflammation and tissue damage in various organs [47–49]. Oxidative stress is defined as excessive production of ROS, including the superoxide anion radical (O_2_^−^), hydrogen peroxide (H_2_O_2_), and hydroxyl radical (•OH); excessive ROS are toxic to the human body [50, 51]. In the present study, the production of ROS and mRNA expression levels of *Il6*, an inflammatory cytokine, were significantly increased under hyperglycemia in rat Achilles tendon-derived cells. Upregulation of inflammatory mediators (e.g., IL1β, IL1, IL6, and tumor necrosis factor-α) strongly suggests that inflammation is a key process in tendinopathy [52]. ROS are generated under the control of growth factors and cytokines by various enzymes, including NOX, and the mitochondrial electron transport chain [53]. Importantly, ROS derived from NOX are important mediators of signaling pathways that regulate crucial physiological activities, such as cell growth, proliferation, migration, differentiation, and apoptosis as well as immune and biochemical responses [54]. This is particularly relevant under pathological conditions; upregulation of tissue-specific and disease-specific NOX subtypes leads to overproduction of ROS [54]. For instance, NOX activation-dependent protein kinase C is thought to play a key role in increasing ROS generation under HG conditions [21]. In fact, Ueda et al. found that HG conditions increased ROS production via upregulation of the mRNA expression of *Nox1* and *Il6* in rat tendons [15]. Additionally, Kurosawa et al. reported that the mRNA expression of *Nox1*, *Nox4*, and *Il6* was higher in rat tenocytes under HG conditions [55]. We found that the mRNA expression levels of *Nox1*, *Nox4*, and *Il6* were also higher in the hyperglycemic state, which agrees with previous reports [15, 55].

Overproduction of ROS has been shown to promote apoptosis, suggesting important crosstalk between oxidative stress and apoptosis [56]. For instance, proapoptotic protein-mediated apoptosis has been shown to be induced by oxidative stress under HG conditions [57]. Thus, hyperglycemia may interfere with the capacity to restore damaged or degenerated tendons. Lin et al. also found that rat patellar tendon cells under HG conditions for up to 48 h exhibited decreases in type I collagen expression, along with increased apoptosis and decreased proliferation (versus cells grown under low-glucose conditions) [58]. In this study, apoptosis induction and ROS production were significantly increased under hyperglycemic conditions. Importantly, the development of such phenotypes was suppressed via quercetin administration.

Gonzalez et al. reported that changes in the morphology and stiffness of collagen fibers (translated into a decreased strain-to-failure of the fibrils) were observed in the tail tendons of Zucker diabetic Sprague–Dawley rats [59]. In another histological analysis, the collagen fibers in diabetic tendons were reported to be poorly organized without changes in the collagen content [60]. Similarly, in the present study, the collagen fiber arrangement in the Achilles tendons of diabetic rats (group C) was significantly disorganized. However, quercetin treatment (group Q) prevented the development of this phenotype. This showed a protective effect of quercetin in the early stage of tendinopathy, but the effect on more chronic and severe tendinopathy has not been proven and needs to be tested in the future.

Type I collagen accounts for approximately 90% of the collagen in normal tendons, whereas type III collagen is mostly found during inflammation [61]. Hyperglycemic conditions inhibit the expression of type I collagen in rat patellar tendons [58]. Similarly, Ueda et al. reported that HG conditions lead to decreased expression of type I collagen and increased expression of type III collagen in rat Achilles tendons [15]. Importantly, we showed that administration of quercetin to diabetic rats significantly increased the expression of type I collagen and decreased the expression of type III collagen in their Achilles tendons (*versus* in diabetic control rats).

The expression balance between matrix metalloproteinases (MMPs) and tissue inhibitor of matrix metalloproteinases (TIMPs) regulates the metabolic activity of normal tendons [62]. Although MMPs cleave damaged interstitial collagen for remodeling in the presence of inflammation, TIMPs control the overexpression of MMPs [62]. Importantly, high glucose levels increase MMP-2 production in adventitial fibroblasts [63]. Additionally, the mRNA expression of MMP-2 and TIMP-2 has been shown to be upregulated in the tendons of diabetic rats [15]. Strikingly, in the present study, quercetin treatment significantly downregulated the mRNA expression levels of both MMP-2 and TIMP-2 in the Achilles tendons of diabetic rats.

However, this study had some limitations. First, we performed preliminary animal experiments; therefore, further detailed studies are needed to understand the potential of quercetin in the clinical context. Particularly, the best method of administration of quercetin to humans (e.g., oral or systemic *versus* local administration) should be determined. Second, application of quercetin in the pharmaceutical field has been limited because its low solubility leads to poor absorption, low bioavailability, low permeability, and instability [28].　However, new preparations of quercetin have emerged in recent years based on nanoparticles [64, 65], polymeric micelles [66], mucoadhesive nanoemulsions [67], and other nanoformulations [68] and were shown to improve solubility and bioavailability. Therefore, further investigations of the effects of quercetin using such formulations are warranted. Third, the difference in the biomechanical properties of the Achilles tendons of diabetic rats subjected to quercetin and no quercetin treatment was not examined because of the limited number of animals. Quercetin has been reported to improve the ultimate stress of healing rat patellar tendons [69]. Therefore, it is necessary to further investigate the effects of quercetin administration on the biomechanics of the Achilles tendons of diabetic rats. Finally, experiments using quercetin inhibitors and other drugs are required to further understand the mechanism underlying the development of our phenotypes.

## Conclusions

Quercetin treatment suppresses NOX expression and ROS production in the Achilles tendons of rats under hyperglycemic conditions. We demonstrated the antioxidant and anti-inflammatory effects of quercetin on the tendons of rats exposed to HG conditions both *in vitro* and *in vivo*. Importantly, quercetin exerted protective effects on the Achilles tendons. Taken together, our results suggest that administration of quercetin in the context of hyperglycemic oxidative stress can prevent the development of diabetic tendinopathy.

## Data Availability

The datasets generated during and analyzed during the current study are not publicly available due to the inclusion of unpublished data but are available from the corresponding author on reasonable request.

## Abbreviations

DM: Diabetes mellitus
ROS: Reactive oxygen species
RG: Regular glucose
HG: High glucose
NOX: NADPH oxidase
DMEM: Dulbecco’s modified Eagle’s medium
DCFH–DA: 2′7’-Dichlorofluorescein diacetate
DAPI: 2-(4-Amidinophenyl)-1H-indole-6-carboxamidine
TUNEL: Terminal deoxynucleotidyl transferase dUTP nick end labeling
SD: Standard deviation
ANOVA: Analysis of variance
